# Some Observations about the Alimentary Canal

**Published:** 1911-06

**Authors:** J. C. Brock

**Affiliations:** Carrollton, Ga.


### SOME OBSERVATIONS ABOUT THE ALIMENTARY CANAL.

By J. C. Brock, Carrollton, Ga.

From the time of Hyporites on down to modern medicine, the conditions of the alimentary canal and its environments have been about the same, both from a physical and pathological standpoint. The improvements made along these lines have come from a better conception of these conditions, and these strides will go hand in hand with other departments of medicine and surgery. The solutions to be made are as difficult or perhaps more so than those which confront us in other departments of medicine. Along these lines, therefore, the doctor fails or makes himself more famous in more instances than in all other departments. It is here that more reflexes mislead us into other organisms than is generally admitted—the heart, the kidneys, the liver, the condition of blood vessels and hundreds of others pains whose cause is often obscured by these reflexes.

So many nitches and impacted sacs in which diseased conditions lurk, producing sectional inflammations, that diagnoses are at times difficult to make. These contentions confront every man, whether specialist or surgeon alike.

The more intelligent along the lines of these alimentary diseases the more we cry out for less harmful causes from food conditions, improper food, improper use of food, bringing to our notice thousands of destroying bacteria from thousands of food sources. The laity cannot settle these food questions, either for the sick or for the individual in health as well.

The intelligent doctor must give shape to the law-making power of the land and see to it that these laws are enforced before the death rate is materially decreased. It is here that more lurking evil invades our homes than from all other sources combined. We are at the mercy of food dealers, among whom are a per cent. that do not hesitate to scatter death and disease broadcast over the land.

The scientific treatment of the stomach and bowels by the modern specialist takes for his sheet anchor the proper diet. The thousands of alimentary nostrums sown broadcast over the land have alike a disastrous influence, inasmuch as they mislead the unscientific into the belief that curative results may follow their use. Thus cheating himself and his patient alike with stuff that often becomes inert in the stomach and may do harm in many cases. Every manufacturer of medicine has his stomach nostrum and all fail from the "rivers to the ends of the earth." The failure is due because the diet treatment was not made paramount. Rest! Rest! Rest! This is the real treatment—the object of everything we do for such cases.

It is not the object of this paper to mention or treat any special disease of the alimentary canal. If it succeeds in making the proper impression of the influence of food, the proper food in diseased conditions, and of the rest period during which the breach may heal of itself, then we feel that its object has been attained.

We all admit the rest treatment for all inflammations, yet many of our doctors fail to observe the proper importance of giving the proper rest to an inflamed alimentary condition. Every sction of the alimentary canal from the mouth through the entire tract, has its pecular inflammations; yet it is a simple inflammation brought on many times from various sources and causes. Yet, top, when these causes are properly looked into we trace them back to improper food or else improper quantity in such proportion that other causes are the exception.

The modern teacher of medicine is giving little or no medicine compared to the importance of the proper diet. The proper diet to alimentary diseases is like clean surgery to its breach. They get well without medicine. What would you think of a surgeon of to-day applying medicine to heal his wounds? It is just as absurd in the disease under consideration, except in acute affections, depending on whatever cause, in which case we remove the cause, whether improper food or the germ life produced by improper food. Here is required the best skill at our command, in which even nature must heal the breach. ducing a perfect digestant for food. The act of nature in its appropriations in most sections of the canal is of itself necessary to the assimilation of foods.

We conclude, therefore, the importance of allowing each section to perform its proper function in order that the mechanism of digestion may be allowed to complete its proper office.

